# Using Normalisation Process Theory (NPT) to develop an intervention to improve referral and uptake rates for self-management education for patients with type 2 diabetes in UK primary care

**DOI:** 10.1186/s12913-022-08553-7

**Published:** 2022-09-27

**Authors:** Jessica Turner, Graham Martin, Nicky Hudson, Liz Shaw, Lisa Huddlestone, Christina Weis, Alison Northern, Sally Schreder, Melanie Davies, Helen Eborall

**Affiliations:** 1 School of Applied Social Sciences, De Montfort University, Edith Murphy Building, The Gateway, Leicester, LE1 9BH UK; 2 The Healthcare Improvement Studies Institute (THIS Institute), University of Cambridge, Cambridge Biomedical Campus, Clifford Allbutt Building, Cambridge, CB2 0AH UK; 3 Department of Health Sciences, University of Leicester, George Davies Centre, University Road, Leicester, LE1 7RH UK; 4 Department of Health Sciences, University of York, ARRC Building, Heslington, York, YO10 5DD UK; 5 Leicester Diabetes Centre, University Hospitals of Leicester NHS Trust, Gwendolen Road, Leicester, LE5 4PW UK; 6 Diabetes Research Centre, College of Medicine, Biological Sciences and Psychology, University of Leicester, Leicester, LE5 4PW UK; 7 NIHR Leicester Biomedical Research Centre, University Hospitals of Leicester NHS Trust and University of Leicester, Leicester, LE3 9QP UK; 8 Usher Institute, University of Edinburgh, Teviot Place, Edinburgh, EH8 9AG UK

**Keywords:** Type 2 diabetes, Self-management, Structured education, Intervention development, Qualitative, Normalisation Process Theory

## Abstract

**Background:**

Referral and uptake rates of structured self-management education (SSME) for Type 2 diabetes (T2DM) in the UK are variable and relatively low. Research has documented contributing factors at patient, practitioner and organisational levels. We report a project to develop an intervention to improve referral to and uptake of SSME, involving an integrative synthesis of existing datasets and stakeholder consultation and using Normalisation Process Theory (NPT) as a flexible framework to inform the development process.

**Methods:**

A three-phase mixed-methods development process involved: (1) synthesis of existing evidence; (2) stakeholder consultation; and (3) intervention design. The first phase included a secondary analysis of data from existing studies of T2DM SSME programmes and a systematic review of the literature on application of NPT in primary care. Influences on referral and uptake of diabetes SSME were identified, along with insights into implementation processes, using NPT constructs to inform analysis. This gave rise to desirable attributes for an intervention to improve uptake of SSME. The second phase involved engaging with stakeholders to prioritise and then rank these attributes, and develop a list of associated resources needed for delivery. The third phase addressed intervention design. It involved translating the ranked attributes into essential components of a complex intervention, and then further refinement of components and associated resources.

**Results:**

In phase 1, synthesised analysis of 64 transcripts and 23 articles generated a longlist of 46 attributes of an embedded SSME, mapped into four overarching domains: valued, integrated, permeable and effectively delivered. Stakeholder engagement in phase 2 progressed this to a priority ranked list of 11. In phase 3, four essential components attending to the prioritised attributes and forming the basis of the intervention were identified: 1) a clear marketing strategy for SSME; 2) a user friendly and effective referral pathway; 3) new/amended professional roles; and 4) a toolkit of resources.

**Conclusions:**

NPT provides a flexible framework for synthesising evidence for the purpose of developing a complex intervention designed to increase and reduce variation in uptake to SSME programmes in primary care settings.

**Supplementary Information:**

The online version contains supplementary material available at 10.1186/s12913-022-08553-7.

## Background

Type 2 diabetes mellitus (T2DM) is a serious, progressive, chronic disease, which often results in reduced quality of life and increased risk of long-term health complications. The incidence of T2DM and its burden on healthcare resources is increasing [1, 2]. There is now greater emphasis on the role of the individual managing their condition [3]. Individuals need information and skills to self-manage T2DM and to make behavioural changes relating to diet, physical activity and medication [4]. In the UK, NICE guidelines recommend provision of structured self-management education (SSME) to individuals with T2DM [5]. This can take the form of group sessions (for example, *DESMOND* [6, 7] or *XPERT* [8]), one-to-one counselling, or other modalities (such as the Diabetes Manual [9–11]), ideally meeting national quality standards [12].

Evidence indicates that SSME is associated with improved T2DM outcomes [6, 7]. However, levels of referral and uptake are relatively low in many countries, including the UK. The addition of a Quality and Outcomes Framework (QOF) indicator for SSME referral for newly diagnosed T2DM [13] has improved GP referral rates in England [14], but substantial variation remains [14]. Frequently, patients do not attend education sessions when offered [15]. Thus, referral to and uptake of SSME for T2DM is inconsistent, for reasons including patient and clinician beliefs about its value, difficulties in accessing sessions, and lack of awareness of provision [15].

The Embedding study (full title: Increasing uptake of effective self-management education programmes for type 2 diabetes in multi-ethnic primary care settings) [16] is a five-year research programme which aims to understand the multi-level influences on this variation, and develop and test an intervention to increase referral and uptake. The aim was not to develop a new SSME *programme*; rather, to develop an *intervention* that would address and improve uptake and referral to existing programmes [16]. The intervention needed to be multi-dimensional to address the breadth of influences on SSME referral and uptake, and target individuals and organisations at different levels, including education providers, commissioners of services, primary care staff and individuals living with T2DM [16, 17].

This paper reports on the development phase of the intervention (the ‘Embedding package’), in line with recommendations for comprehensive and transparent reporting on the development of complex interventions [18]. Details of the feasibility and full trial of the resulting intervention are reported elsewhere [16, 17]. Recognising that theory-informed interventions are more effective than those not informed by theory [18–20], we chose to draw upon Normalisation Process Theory (NPT) [21] as a constructivist analytical lens through which to approach, organise and assimilate evidence to understand key issues in the implementation of SSME. These included how and why different stakeholders ‘buy in’ (or not) to SSME, and the issues that an Embedding intervention would need to address to increase likelihood of implementation, routinisation and sustainability [21]. Thus, it is likely to have broader methodological utility for the development of complex interventions in theoretically and empirically informed ways.

NPT contends that complex interventions only become integrated into existing practice through a conjoint process of normalisation at individual and collective levels [22]. It focuses on the meaning that participants in the implementation process attribute to new interventions, and the work they do individually and collectively to implement and embed it in everyday practice (or to contest, resist or disrupt implementation) [21–23]. ‘Participants’ include any individuals or groups involved in or impacted by an intervention – e.g. receiving, delivering or commissioning it. NPT proposes four constructs that explain how participants approach implementation of a new practice: coherence (making sense of the innovation/intervention); cognitive participation (engagement with it); collective action (work done to enact it); and reflexive monitoring (appraisal of it) (see Supplementary Table 1) [21, 24]. These constructs provide a way of investigating and understanding the dynamics of implementing, integrating and sustaining a healthcare intervention [21, 24]. It has also been argued that NPT might offer a basis for developing interventions that are more likely to be implemented and sustained successfully [25], and the flexibility of the framework made it particularly appealing as a methodological tool with which to approach the development of an intervention to address referral and uptake. However, our recent literature review demonstrated that despite NPT’s potential for informing an intervention’s *development*, it is much more commonly used to *evaluate implementation* of an intervention [26]. To our knowledge, there are only two examples of use of NPT to develop interventions in primary care [27, 28]. A secondary aim of this paper, therefore, is to provide an exemplar model for using NPT to inform development of a complex intervention in primary care. Cognisant of the importance not only of theory-informed intervention development but also development that engages with and benefits from the expertise and experience of those involved in implementation, such as clinicians, patients and other ‘end users’ [29], we sought to involve these groups in various ways throughout the process. We describe our approach in more detail in the next section.

## Methods

### Aim

The aim of the development phase of the Embedding study was to collaboratively develop an intervention to improve uptake and referral to existing SSME programmes for T2DM in UK primary care. Our objectives were to: 1) identify attributes of an embedded (i.e. routinised and normalised) SSME programme; 2) refine and prioritise these attributes within the current organisational context of UK primary care; 3) identify the key components that a successful intervention would require.

### Design

**Fig. 1:**
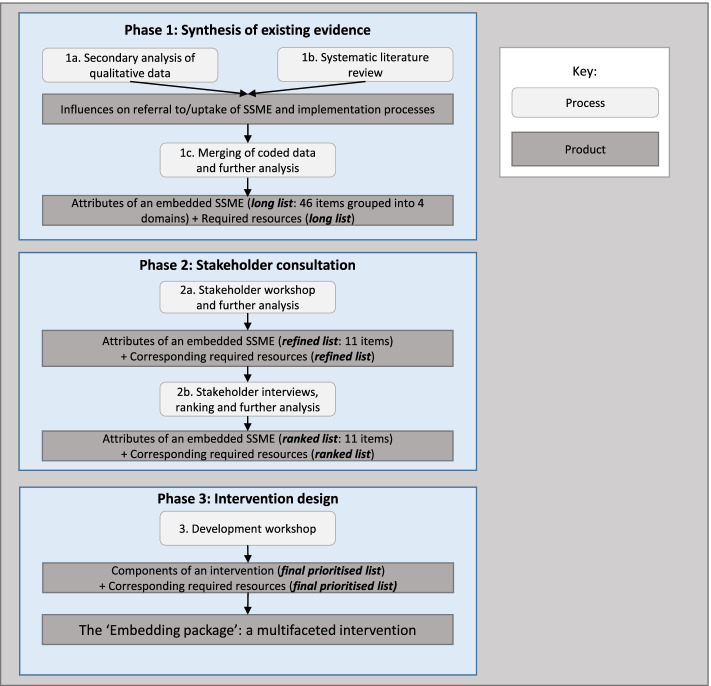
The iterative design process of the Embedding study intervention

We took a phased, iterative approach to intervention design and development, drawing upon NPT to guide analysis in the first two phases (see Fig. 1 for an overview; phase 1: Synthesis of existing qualitative data and published literature; phase 2: stakeholder consultation; phase 3: intervention design). The resulting intervention had to be ready to test in a feasibility trial that formed the next stage of the Embedding study [17].

### Phase 1: synthesis of existing evidence

This phase included three parts: secondary analysis of existing qualitative datasets (1a); a systematic literature review (1b); and an integrative analysis bringing the two together (1c). The first two parts of phase 1 were conducted in parallel. Our analytical approach for all three parts drew on the framework method [30, 31], incorporating constructs from NPT [24]. The framework method stages include: familiarisation with data, coding, developing a working analytical framework, applying the framework, charting data into the framework and interpretation [30, 31]. Notably, the framework method allows coding to pre-defined categories (e.g. relating to existing theory) [31]. In the following section we describe our analysis in more detail.

#### 1a. Secondary analysis of qualitative data

Members of the Embedding study team had previously gathered qualitative data about various SSME programmes [32–37]. Collectively these data promised an untapped source of knowledge about enablers and barriers to uptake; undertaking a secondary analysis of the combined datasets offered an efficient method for assimilating these. The initial sample comprised eight datasets of interviews and focus groups (147 transcripts), covering the views and experiences of participants in SSME programmes, programme deliverers, and primary care staff (see Table 1 for details). We sampled within these datasets to produce a more focused subset of transcripts, pertinent to our aim. To do this, we first read a selection of transcripts and reviewed the topic guide from each dataset, noting content relating to uptake of or referral to SSME (for example needs and preferences; experience of a specific SSME programme). This process initially led to the exclusion of three datasets where the data focused on specific features of certain interventions, rather than on SSME more generally.

**Table 1:** Details of the qualitative datasets available for sampling for Phase 1a

Study (in alphabet order of short title)	Setting	Data collection method	Participant demographics	Included in final sample?
DESMOND Foundation study [28]	Leicester & Birmingham	Individual semi-structured interviews (in person)	19 adults with established T2DM: 9 female, 10 male; 12 BAME, 7 White European; age: 43–83 years, median 59	Dataset excluded after initial coding stage
DESMOND Lay Educator study (staff) [29]	Four sites across England and Scotland	Individual semi-structured interviews (telephone)	11 SSME Educators: Healthcare professional SSME educators [6], lay SSME educators [5]	Dataset excluded after initial coding stage
DESMOND Lay Educator study (participants) [29]	Four sites across England and Scotland	Individual semi-structured interviews (telephone)	16 adults with newly-diagnosed T2DM: Female 11, Male 5	Dataset excluded at screening stage
**DESMOND ongoing study (staff)** [30]	**Leicester & Sheffield**	**Individual semi-structured interviews**	**27 transcripts:** **SSME Educators** [11]**, practice nurse (*n* = 5), GP (*n* = 3), research team** [8]	**19 transcripts (SSME Educators and primary care staff)**
**DESMOND ongoing study (participants)** [30]	**Leicester**	**Individual semi-structured interviews**	**29 transcripts:** **31 adults with established T2DM (2 paired interviews)** **Female 14, male 17; Age: 29–87 years, median: 68**	**29 transcripts**
DESMOND self-monitoring study (staff) [31]	Leicester & Cambridgeshire	Focus groups and individual interviews (telephone)	11 transcripts (4 focus groups, 7 interviews) 23 SSME educators	Dataset excluded at screening stage
DESMOND self-monitoring study (participants) [32]	Leicester & Cambridgeshire	Individual semi-structured interviews (in person)	18 adults with newly diagnosed T2DM 7 female, 11 male; age: 29–80 years	Dataset excluded at screening stage
**Programme Development Grant** [33]	**Leicester & Cumbria**	**Individual semi-structured interviews (telephone and in-person)**	**16 transcripts:** **Commissioners (*n* = 3), GPs (*n* = 3), practice nurses (*n* = 3), practice managers (*n* = 2), SSME educators (*n* = 2), SSME coordinator (*n* = 1), community health development workers (*n* = 2)**	**16 transcripts**

*Note*: Datasets presented in bold were included in the final sample

We developed a coding framework by adapting generic sensitising questions pertaining to NPT’s constructs as advocated by May and Finch [21], into questions specific to SSME and the primary care setting (see Table 2 for the coding). LS coded three transcripts from each of the five remaining datasets, according to these thematic headings. Discussion of the coding findings at this stage (LS,JT,HE,NH,GM) led us to focus on three datasets, which we imported into qualitative data-indexing software. LS coded the final included sample (64 transcripts) according to the NPT-informed framework, meeting regularly with JT to refine the framework in light of insights arising inductively from the data.

**Table 2:** Sensitising questions (structured by Normalisation Process Theory constructs) that informed the coding framework

Construct	Interview transcripts coded against these questions^a^	Full text articles coded against these questions^a^
Coherence	What are individuals’ attitudes to self-management and SSME? Is it different from other interventions? What is the potential value, benefit and/or importance of SSME?	What enables understanding and differentiation of the intervention? What are the barriers to understanding and differentiation of the intervention?
Cognitive Participation	What are the barriers to self-management? What are the barriers to uptake of SSME? What is their motivation for participating?	What enables individuals and groups to engage with the intervention? What inhibits individuals and groups from engaging with the intervention?
Collective Action	Are the right people running it with the right skills? Do individuals trust the intervention to work? How do people make it work? How does it work? Is it supported by management, policy and/or resources?	What helps individuals and groups to undertake the work of the intervention? What are the barriers to individuals and groups undertaking the work of the intervention?
Reflexive Monitoring	Have individuals made changes as a result of it? How do they know it works? Have they any suggestions for improving it?	What enables individuals and groups in understanding and evaluating the work of the intervention? What inhibits individuals and groups from understanding and evaluating the work of the intervention?

^a^ Questions informed by May and Finch [21]

#### 1b. Systematic literature review

The focus of the literature review was the application of NPT in informing and assessing implementation processes in UK primary care settings. We were interested particularly in: what types of UK primary care interventions use NPT; how NPT was operationalised in these examples; how authors reflect on the use of NPT; and ultimately what this could tell us about attributes of an embedded intervention in UK primary care.

A full description of the methods for searching and extracting data for this review has been published elsewhere [26]. In brief, seven bibliographic databases were systematically searched for terms relating to primary care and NPT, followed by hand-searching of reference lists contained in included articles to identify any additional relevant articles. Data were extracted by JT and LS, using a data extraction form to collate study characteristics (design, methods, sample, setting, topic and implementation stage) to determine eligibility for inclusion. Twenty-three eligible full-text articles were imported into NVivo for analysis [38–60]. JT used a similar coding framework to that employed in phase 1a to code excerpts from the publications (Table 2), meeting regularly with LS to discuss and refine the framework.

#### 1c. Merging of coded data and further analysis

From the combined coded dataset, we reviewed all data coded to our NPT-based frameworks (Table 2) to produce a comprehensive list and descriptive summaries of *influences on uptake of and referral to SSME programmes* and the *implementation processes* involved. In a team analysis meeting (JT,LS,NH,GM,HE), we worked systematically through the list, first reframing all items as *desirable attributes of an embedded SSME system*, and then exploring the interrelationships between items, grouping them into four overarching domains. This process ensured that our preceding analysis was organised accessibly for the next phase of the work, to enable consideration of how they might best fit together and to prioritise them. AN and SS used the list to produce a complementary list of *the resources required to realise each attribute*.

### Phase 2 – stakeholder consultation

In phase 2, we sought input from wider stakeholders to ensure that our analysis and its implications for intervention (in terms of desirable attributes and resources required to achieve them) were relevant to the current organisational context of primary care. This ensured that the social-scientific perspective provided by the core team was complemented by the views of other relevant stakeholders, including the trial team, patients and the public, and healthcare professionals. This involved two stages: a stakeholder workshop, then stakeholder interviews and ranking.

#### 2a Stakeholder workshop

We recruited a range of stakeholders with differing expertise in relation to T2DM in primary care to the workshop; we invited all Embedding study co-investigators, wider research team members including the Patient and Public Involvement (PPI) Lead, and additional healthcare and research staff associated with the study and/or other relevant studies or clinical practice linked to the host institution. Using a focus group format, we provided cards printed with each identified *attribute of an embedded SSME*. First, we facilitated the discussion using a semi-structured format that involved asking participants to consider each attribute and whether it made sense, its feasibility in ‘real world’ primary care, and its importance. Participants were invited to discard attributes they considered unimportant, add any missing attributes, and group associated attributes together. Next, we invited discussion of the resources required to realise the attributes that were seen as most plausible, feasible and important in the initial discussion. We used the list of resources produced by AN and SS to provoke discussion, but also encouraged brainstorming of further potential resources. We captured data by audio-recording discussions, taking written notes throughout and photographing the arrangements of cards.

In an analysis meeting (JT,LS,NH,GM,HE) after the workshop, we drew upon these data to annotate the list of attributes, by discussing key themes from the discussion around each attribute, then combining, amending, adding and removing items into a shortlist, with accompanying explanatory notes. This process gave rise to a shortlist of eleven priority attributes of an embedded SSME, and a corresponding compendium of resources required to realise an embedded SSME system (including pre-existing resources, resources requiring further development and new resources).

#### 2b Stakeholder interviews and ranking

Given the need for a manageable and replicable intervention, we sought to narrow down the shortlist of attributes. We engaged a further set of stakeholders with no previous involvement in the study: professionals with an interest in SSME commissioning, referral or delivery. We used two routes of engagement: direct invitation to an individual interview targeting stakeholders identified by the Director of the DESMOND SSME Programme; and a broader consultation exercise with primary care professionals attending a ‘Diabetes Update’ meeting, hosted by a local diabetes education network.

For individual interviews, we invited stakeholders by email, attaching a participant information leaflet. Those who replied indicating interest were sent a consent form and ranking exercise: a list of the attributes shortlisted during Phase 2a with instructions to rank them in order of priority from most to least important. Interviews were semi-structured in format; the topic guide was informed by the NPT constructs and shortlisted attributes and questions covered: experience of SSME; which attributes they had ranked as most and least important and why, and which they struggled to rank; and what format a potential intervention could take and what it might include. All interviews were conducted by phone and audio-recorded with participants’ consent.

For the broader consultation exercise, part of the Diabetes Update meeting was set aside for a structured discussion. NH provided delegates with the project background and invited delegates to ask questions. Paper copies of the ranking exercise used in the interviews were distributed to delegates. Participation was optional; those who participated provided signed informed consent.

We used the Borda ranking approach to create an overall ranking of attributes [61], collected through both routes, and drew up on the framework method to analyse accompanying explanatory qualitative data [30, 31].

### Phase 3: intervention design

The aim of Phase 3 was to move from the ranked list of attributes, and corresponding resources, into identifying and developing key components of an intervention, the ‘Embedding package’. Recognising the need to focus on the attributes seen as most likely to influence implementation, given the finite resources of the team and the need to provide a clear steer for intervention design, we focused on the attributes ranked most highly in the Borda process.

In a team meeting (JT,LS,HE,NH,GM,AN,SS,MD) we discussed the ranked list and accompanying qualitative findings from Phase 2 and agreed to focus on the top five ranked attributes, which, from this point onwards, became *key components of the intervention*.

Fortnightly meetings (JT,LS,AN,SS,MB) ensured the data collected in Phases 1 and 2 directly informed the development of the intervention to be implemented in the feasibility trial [17]. A corresponding process focussed on mapping existing resources onto the key components and identifying existing resources which needed further development and resources which did not yet exist to support the components of the intervention effectively.

## Results

### Phase 1: synthesis of existing evidence

#### 1a. Secondary analysis of qualitative data

The final sample of transcripts from the three selected datasets included 31 recipients of SSME, 30 frontline delivery staff (13 SSME educators, one SSME coordinator, six general practitioners, eight practice nurses, two community development workers) and three commissioners. Here, we briefly summarise key findings from the secondary analysis under NPT’s four constructs. Table 3 gives examples of coded data to illustrate this.

**Table 3:** Examples of data extracts coded to NPT constructs

NPT constructs	Selected example data
Coherence	*Examples of views about whether or not SSME was seen as distinct from routine care* • “It was different from previous reviews with the GP—it included action planning and goal setting.” (Recipient; DESMOND Ongoing; ID 1.01.030) • “The feedback I got back from patients was fantastic. They all really loved it. […] They found it so helpful. It was the first thing really that people had ever had access to so we were very keen to get DESMOND up and running here because there was not very much that people could access in [location]” (Practice nurse; DESMOND Ongoing; ID PS-A-021) • “I don’t think it was that different really. I mean I think we do really try and give people responsibility. Plus we do have a lot of support because we have a diabetes specialist nurse who will come in and also give people support”. (GP; DESMOND Ongoing; ID PS-B-023)
Cognitive participation	*Buying into the idea of SSME and what it involves* • “I would like to see more GPs come to observe it […] I have had one or two practice nurses [observe], some do but again it’s time constraints. But I have never had a GP […] I personally think that would help them and some commissioners to see what it’s all about.” ([role?], PDG, 020,101) • “I think the two primary factors behind [low referral] are possible ignorance as to what is involved in the process and secondly lack of local resources so that if you refer and the patient has to wait for weeks or months, which is locally our case, they [don’t attend] and therefore don’t engage in the process and there is nothing coming back to us about the useful impact of SSME. (GP; DESMOND Ongoing; PS-B-019) • “It pushed you to think more about how you can take responsibility and help yourself & to set goals” (Recipient: DESMOND Ongoing; ID 1.01.024) • “I am self-employed so when work comes in you have to do the work. You know these things [SSME] take a long time and you have got to take a day off work so it costs me a day’s money […] so it has to be a relative benefit and to be honest after the first ones that I went to, I was not getting any benefit from them at all.” (Recipient: DESMOND Ongoing; ID 1.01.029)
Collective Action	*Debates about funding and delivery of SSME* • “Time and resources would be need to implement [SSME] in primary care… you couldn't fit these activities into a normal practice”. (Educator; DESMOND Ongoing; ID ES-A-017) • “I think the fact that practices are seeing diabetics, the nurses are spending quite a lot of time with patients already, it probably would be a better model if they were formally trained to deliver [SSME], partly on a one-to-one and some of it in a group in the practice. I think that would be a far more cost effective way of doing it. And probably better because the people who are providing the ongoing care, if you like are brought into those messages as well. I think it’s a far better model rather than sending someone on a [course].” (Commissioner; PDG; ID 020,104)
Reflexive monitoring	*Views about effectiveness and impact of SSME and suggestions for improving it* • “[SSME] needs to be constantly evaluated to be cost effective”. (Frontline Delivery Staff, PDG, 020,101) • “Needed to be prepared and to have done some thinking before the care planning appointment, so that you are not put on the spot and come up with goals that you are pulling out of thin air, needs to be explicit that people need to be prepared”. (Recipient; DESMOND Ongoing; ID 1.04.005) • “I suppose it is too expensive to tailor it to each person, but maybe have different options for people because people are at different stages [of T2DM duration]. People who are retired can perhaps attend any time. They might have different barriers of access or being able to get somewhere or health problems or just simply not being able to drive, but then you get the younger people who are working five days a week and […] it is a drain on resources to keep having half days off.” (Recipient; DESMOND Ongoing; ID 1.01.067)

In terms of *coherence* and making sense of SSME, the majority of recipients and commissioners acknowledged its value both for people with T2DM and staff providing their care, and could distinguish between the role of SSME and routine care. However, some frontline delivery staff were unable to differentiate the two: one of the reasons why referral to SSME may be inconsistent.

Data coded to *cognitive participation* revealed logistical and organisational influences on making SSME happen within routine practice, including the need for more effective communication and joined-up working between SSME providers and practice staff, given many conflicting demands. While some recipients readily ‘bought in’ to SSME and the work required from them in self-managing, barriers to uptake included, for example: the challenge of fitting SSME provision into their existing commitments (e.g. working, caring for family); perceived stigma; religious, cultural and linguistic barriers; and physical and other access difficulties.

Views were mixed in relation to *collective action;* arguments for in-house SSME delivery by practice staff cited continuity of care and efficiency, but were countered by the scale of investment required and need to balance scarce time and resources at practice level, as well as acknowledging the specialist skill of external SSME Educators.

Concerning *reflexive monitoring,* while reports of recipients who had benefited from SSME were plentiful, many staff and commissioners favoured continuing evaluation of SSME provision. Suggestions for improvement included: greater integration of SSME into routine primary healthcare; tailoring and adapting SSME provision to local needs (in terms of access, working patterns, culture and language); and flexibility of different modalities of delivering SSME, including a combination of approaches (e.g. ‘homework’ to aid preparation for SSME sessions).

#### 1b. Systematic literature review

Twenty-three articles were identified during the literature search. Key findings of the review pertinent to development of the Embedding package are summarised here^1^; for a more extensive account, see [26]. Supplementary Table 2 provides examples of excerpts from included papers coded to each NPT construct.

A key theme was the importance of *coherence* and understanding the sense-making people do about interventions in primary care, including conceptual coherence and the practical work involved in implementation. Successful implementation was often associated with the existence of a sound evidence base for an intervention, careful timing, and alignment with current policy and guidance. Other factors included the intervention’s purpose, its distinctiveness from existing practice, and the adaptability of existing practice to accommodate the particular intervention [39, 40, 42–45, 49, 55, 56, 59].

In terms of *cognitive participation*, examples of relational work done in the course of the normalisation of new interventions in routine practice were efforts to ‘fit’ an intervention to the incumbent organisational context, including existing professional and patient roles and wider policies, pathways and processes. Key features of successful implementation included user involvement in the design and championing of an intervention [39, 40, 43, 44, 49, 57, 59].

Among the most important influences on *collective action* and the operational work undertaken by participants in implementation processes were the sensitivity of an intervention to local context, and how easily it could be adapted to local circumstances. Sufficient resources and support were also important, as was the visibility of the intervention to relevant stakeholders at all levels [39, 40, 43, 44].

Several papers highlighted the importance of *reflexive monitoring* and the robust recording, auditing and evaluation processes for appraising, sustaining and continually adapting interventions. Tools to enable regular user feedback and allow structured reflections to be incorporated into the implementation of the intervention were also seen as important [39, 43–45, 49].

#### 1c. Merging of coded data and further analysis

A ‘longlist’ of 46 *desirable attributes that would characterise an embedded form of SSME*, resulting from the merger of influencing factors identified in phases 1a and 1b, is provided in Table 4. The attributes mapped into four overarching domains; that the SSME be: 1) **valued** (by relevant stakeholders and systems), 2) **integrated** into local primary care systems (and supported with resources and staffing), 3) **permeable** (accessible and promoted) and 4) **effectively delivered** (tailored and flexible, while remaining consistent and monitored); for full details of each domain, see Table 4. This constituted a crucial juncture in the intervention development process, as it represented the point at which we moved beyond an analytical framework explicitly informed by NPT, and sought instead to frame our findings in terms of the specific empirical field and focus of the intended intervention, i.e. the uptake of SSME in an English primary care setting. The four domains came from our discussion about the attributes and sought to cut across NPT’s constructs to frame the desirable attributes of the intervention in terms of *empirical traits to be operationalised in a specific intervention*, rather than *conceptual constructs relevant to the normalisation of all interventions*. In this way we sought to make the desirable attributes accessible to wider stakeholders involved in phase 2.

**Table 4:** Attributes of an embedded intervention mapped onto four domains

	Attributes
It is **valued**: Intervention is supported by evidence Distinct from but not at odds with current practice Staff need to understand/see the value Confidence/trust in intervention Assists with role Fits QOF, wider policy, regulation etc Fits NHS pathway	• Cost effective • Demonstrable clinical and quality of life outcomes • Demonstrable relevance to other NHS services • Based on evidence and academic freedom • Aligned with national & local standards of care • Incorporates evaluation & auditing • Accreditation fits existing models • Examples of good practice are disseminated • Potential benefits and staff’s achievements in using the intervention are celebrated and communicated via announcements, newsletters, and e-alerts • Champions volunteer to undertake role • In addition to recruiting enthusiasts, sceptics are also recruited, working with the developers until their needs are met and are convinced of the value of the intervention
It is **integrated** into local systems: Integrated/joined up systems Time to do it Support materials Practice staff are trained Central leadership & coordination Monitoring & evaluation built in There is follow up and support afterwards	• Availability of referrals & booking systems for practice staff • Collaboration between departments is fostered & maintained in order to maximise the potential of the intervention • Potential to be used for other chronic conditions • Employment of clinical champions and community advocates • Creation of links to community activities and venues • Quality assurance criteria are adhered to throughout • Different elements of the intervention (e.g. content, pedagogy and technology) work in unison • Prominent agenda item at high level meetings • Time for staff to master the intervention • Practice staff awareness of what the intervention offers and does • A strong commitment is needed from the practices and sites in terms of strategies, plans and processes to support and upskill staff • Provision of on-going support for staff • Provision of free resources • Provision of access to appropriate, reliable and future proofed equipment • Build time for delivering the intervention into staff job plans • Provision of follow on care and advice • Integration with diabetes care
It is **permeable**: Awareness exists Provision is tailored to local context Access is individualised Communication with recipients is effective	• Accessible in a number of ways • Involves wider support network [than the patient] where appropriate, including partners, parents, children and carers • Delivered by practice staff who can develop an ongoing relationship with recipients and at a local, accessible and familiar venue • Recipients should be able to drop in and out as required • Flexible to patients, practices and sites, in terms of being tailored to local needs • Adaptable to the needs of different individuals and communities • Group sessions should be arranged for peer groups (e.g. similar age/background/culture/fitness levels)
It is **effectively delivered:** Content is tailored appropriately Delivery is flexible Consistent content & messages throughout	• Delivered in residential & care homes • Available in a variety of formats/languages • Style & delivery is adapted to meet the needs of individuals • Developed and delivered in respect of copyright rules • Provision of easy to use with navigational tools and supporting material (e.g. guidelines) • Associated resources are coordinated and shared to maximise efficiency • Regularly modified and kept up to date • Developed and led by those who use it, user piloting and feedback is crucial • Implement systematic procedures for obtaining staff input • Problems are addressed with quick solutions • Continuity of care & delivery (i.e. by the same people)

Table 5 gives examples of this process of translating influences on uptake and referral and implementation processes into attributes. Table 6 shows examples of how the attributes were mapped to a list of the resources required to realise each attribute.

**Table 5:** Examples to demonstrate how coded data mapped into resulting attributes

*NPT constructs*	*Key influences on uptake of / referral to SSME programmes and/or key implementation processes*	*Attributes of an embedded SSME*
*Coherence*	Stakeholders understand what the intervention is and are able to distinguish the intervention from other initiatives and from routine care	Practice staff awareness of what the intervention offers and does
*Cognitive participation*	Key individuals initiate and/or support the implementation	Employ clinical champions and community advocates
*Collective Action*	Sufficient resources and support are provided by the organisation in which the intervention is implemented	A strong commitment is needed from the practices and sites in terms of strategies, plans and processes to support and upskill staff
*Reflexive Monitoring*	Robust recording, auditing and evaluation processes are in place	Incorporates evaluation & auditing

**Table 6:** Examples to demonstrate the mapping process from attributes to resources

*Attribute*	*Resources—Existing*	*Resources – Needs adapting*	*Resources – Wish list*
Availability of referrals & booking systems for practice staff	• Example referral pathways – ability to tailor to local population • Sample letters & forms • Scripts & guidance for phone calls to patients & reminders	• Training package & resources for referrers • Guidance for the ‘sell’ • How to run a referrer engagement event • Tent stands for desks in clinics • Guide on working with local communications teams • Strategy/ideas for promotion to stakeholders at all levels (patients, community awareness, healthcare professionals and referrers)	• Admin system • Easy to book referral system • Flagging system (patient)
Flexible to patients, practices and sites, in terms of being tailored to local needs	• Guidance on culturally adapting programme	• Guidance on ensuring practical accessibility to groups – re times/locations/venues/ transport/room type • Tailoring groups for minority groups (mental health, intellectual disabilities, nursing homes, etc.)	• Healthcare professional awareness campaign
Associated resources are coordinated and shared to maximise efficiency	• eLearning • Scripts/Guidance for phone calls to patients & reminders • Sample letters & forms	• Training package and resources for referrers	• Easy-to-book referral system, tailored to local population (and linked to administration system) and web-based • Administration system

### Phase 2: stakeholder consultation

Phase 1 of the development process allowed us to draw on existing evidence and theory to identify the necessary attributes for successfully embedding SSME. However, the analyses in phase 1 only generated a list of attributes; it did not involve attempts to organise them into a viable intervention, and nor did it provide active stakeholder involvement in the development process. In phases 2 and 3, we sought to address these objectives, beginning with stakeholder involvement to prioritise the desirable attributes.

#### 2a. Stakeholder workshop

Key themes from the 13 stakeholders’ discussions at the workshop included: efficient and easily accessible referral process to SSME for all staff in primary care; tailoring the SSME to different patients’ needs and making it accessible to all; high quality, effective and cost-effective delivery and evaluation; high quality patient resources; widespread awareness to primary care staff and potential participants including communicating the evidence. Our analysis of the themes led to a condensed list of eleven priority attributes of an embedded SSME (see Table 7) and a shortened and refined corresponding compendium of resources.

**Table 7:** 11 priority attributes of an embedded SSME

*Priority attributes*	*Summary of explanations*
A. Effective referral processes and booking systems	Practice staff need a user-friendly system for identifying candidates for SSME and referring them. Effective professional-patient communication about the referral process ensures that staff, patients and those involved in the delivery of the SSME each know how to use this system and what to expect
B. SSME is tailored to a range of audiences	Ensuring that the SSME is culturally and linguistically appropriate and that an individual’s learning needs and preferences are taken into account when delivering SSME is key for patient buy-in
C. SSME is effectively delivered	SSME programmes must meet national and local strategies, policies and regulations, including NICE requirements, have a structured and written curriculum, be delivered by trained educators, and quality assured and audited
D. SSME is aligned with national & local standards of care	
E. Wider awareness about SSME with primary care staff	All staff within a practice must understood what SSME is, including content and delivery style; meaning they can answer patients’ questions and provide relevant information. Staff with a role of championing SSME would be useful in promoting awareness
F. Wider awareness about SSME with patients & the public	Visibility and availability of SSME can be publicised to potential recipients at practice level – both in materials on display (e.g. posters/leaflets) and within consultations – and in public settings (e.g. gyms or via the media)
G. High quality resources and information for patients	Patient-facing information about SSME must be clear and effective, including information provided prior to attending, as well as information to take away from a session or to access via the internet
H. SSME is accessible for patients	Efforts are needed to address barriers to access in order to make SSME accessible to anyone with T2DM
I. High quality evaluation & auditing	Capturing regular feedback from recipients and staff can inform flexibility and tailoring, as well as identification of any problems. Auditing national databases will provide key quantitative data
J. SSME is cost effective	Above all other considerations, ensuring, improving and demonstrating cost-effectiveness, for all stakeholders is vital
K. Communication about the efficacy of SSME to all stakeholders	Communicating evidence to all stakeholders about how and why SSME could improve health outcomes in the short and long term is key

From the second part of the stakeholder workshop, which focused on the resource requirements, stakeholders drew upon their varied experiences to add further items and insight to the longlist of resources (from Phase 1c). For example, existing resources identified as useful included: patient testimonials; business cases for providers tendering; guidance on carrying out needs assessments; guidance on culturally adapting SSME programmes; sample letters and forms (such as patient invitations or employer letters); example referral pathways to tailor to local populations; eLearning; job descriptions (such as local coordinator, lay educators); and scripts/guidance for phone calls to patients. Suggestions were made for improvements to existing resources. Examples of proposed new resources that could be designed included: commissioning model guidance (such as ‘What kinds of interventions can I buy to address this need?’, ‘How much will they cost?’, and ‘What monitoring information should be required from providers?’); high-quality administrative systems (such as easy-to-book referral systems, systems for recording patient details and SSME details, tracking and reporting referral and attendance); and clearer descriptions of SSME.

Existing and ‘new-build’ resources needed to support the intervention were collated in a web-based application, Trello, for discussion, review and refinement by the research team.

#### 2b. Stakeholder interviews and ranking

The 16 stakeholders interviewed included commissioners, healthcare practitioners (GPs, practice staff, nurses), SSME providers and educators, researchers, and representatives of national diabetes charities. The results of the interviewees’ rankings are presented in combination with the larger group who participated in ranking (Table 8).

**Table 8:** Results of Borda ranking exercise

*Order*	*Priority attributes Statement*	*Score*
**1**	**E. Wider awareness about SSME with primary care staff**	**187**
**2**	**A. Effective referral processes and booking systems**	**163**
**3**	**B. SSME is tailored to a range of audiences**	**159**
**4**	**H. SSME is accessible for patients**	**157**
**5**	**F. Wider awareness about SSME with patients & the public**	**148**
6	D. SSME is aligned with national & local standards of care	145
7	C. SSME is effectively delivered	138
8	G. High quality resources and information for patients	133
9	J. SSME is cost effective	110
10	K. Communication about the efficacy of SSME to all stakeholders	103
11	I. High quality evaluation & auditing	70

Findings from the interviews reinforced findings from analysis in previous phases (e.g. the importance of raising awareness of SSME; the role of practice staff; debates about practice resources and responsibility for SSME delivery; the importance of partnership working; and challenges of ensuring accessibility). In addition, interviewees made suggestions for the concrete form an intervention might take, including a project management tool to enable oversight and coordination of the process from commissioning to delivery, with portals to enable access to appropriate supporting resources.

Forty-two participants (16 interviewee participants and 26 delegates at a Diabetes Update Meeting) participated in the ranking exercise; the resulting Borda rankings are shown in Table 8. Analysis of the interviews and Borda rankings were presented to the full trial team to finalise the key components of the intervention. While drawing on all 11 attributes, the team paid particular attention to those ranked as most important, including: the need for increased awareness about SSME among primary care staff (attribute E) and patients and the public (attribute F); the need to improve referral processes and booking systems (attribute A); the need to tailor SSME to a range of audiences (attribute B); and the need to increase accessibility to SSME for patients (attribute H).

### Phase 3: intervention design (the ‘embedding package’)

Our discussion about the top five ranked attributes (those seen as most likely to make the biggest difference to the routinisation of SSME at multiple levels of practice) and the corresponding list of resources, translated into four key components:

1. A clear marketing strategy for SSME (particularly addressing priority attributes E and F: wider awareness of SSME among primary care staff and among patients and the public)
2. A user-friendly and effective referral pathway (particularly addressing priority attributes A and H: effective referrals and bookings systems, and accessibility for patients)
3. New/amended professional roles (particularly addressing priority attribute E: wider awareness of SSME among primary care staff)
4. A toolkit of resources (addressing all priority attributes, but particularly B: tailoring SSME to a range of audiences)

To put these components into practice, we designed a multifaceted intervention – the ‘Embedding package’ – that combined new leadership roles and pathways for SSME uptake and referral with a web-based portal that offered easy access to key resources. At this point, the team responsible for designing and delivering the intervention (SS,AN) took the lead. A full description of the Embedding Package is reported elsewhere [16, 17]. In brief, it comprised two new roles, the ‘Embedder’, and a local clinical champion (undertaken as part of existing roles in each participating CCG) and an online toolkit.

The two roles were designed to work together to build and sustain buy-in at all levels of local organisations and with all key stakeholder groups, for example: working with primary care teams on timely referrals and good practice; working with SSME providers to review and advise on existing systems and processes to improve uptake or dropout rates to SSME; and promoting SSME to healthcare professionals in the local area and assisting with the development of a locally appropriate referral pathway. A key task for the Embedder was to ensure that referrers and providers were aware of and accessed key elements of the online toolkit.

The online toolkit was designed to be a user-friendly portal to resources for all relevant stakeholders. It consisted of three sections: ‘How to guides’ providing a wide range of strategies for increasing patient attendance at SSME, such as carrying out a needs assessments and working with PPI groups to adapt programmes; a ‘Promoting to patients’ section with tools for designing and implementing marketing and communications plans; and an ‘Increasing referrals’ section that detailed activities to strengthen the referral process, such as engagement events and evaluation of existing referral pathways and administration systems.

Finally, as part of the embedding package, and supported by the Embedder role, stakeholders across the pathway, including patients, had access to a prototype online SSME programme for individuals with T2DM. This online programme complemented, rather than replaced, attendance at group-based SSME.

## Discussion

Through an iterative process involving synthesis of existing data and stakeholder consultation, and informed by NPT, we designed an intervention, the ‘Embedding package’, which aimed to improve referral to, and uptake of SSME for T2DM in primary care. This multifaceted intervention consisted of two new roles and an online toolkit designed for all stakeholders. We highlight key features of our approach to using NPT to inform intervention development, and reflect on the strengths and limitations of our approach more broadly.

### Using NPT to inform intervention development

We selected NPT to inform our approach to intervention development because it offers a flexible framework for analysing how and why different stakeholders ‘buy in’ to the idea and realisation of an innovation (in this case, SSME), as well as providing an understanding of the contextual issues that need to be addressed to enhance implementation, routinisation and sustainability [23, 24]. When we began, NPT had more commonly been used to *evaluate* implementation of an interventions (see [26]) so there was no exemplar to guide our application to intervention *development*. In practice, we found NPT to be a useful analytical lens through which to approach, organise and assimilate pre-existing data to understand key influences on referral to and uptake of SSME and implementation processes in primary care, particularly in phase 1 where we sought to synthesise evidence from a number of sources. It ensured comprehensiveness of scope of secondary analyses of data and literature, guiding our focus towards influences on implementation and how they might be transferable to other settings; for this early work in particular, it offered a framework that ensured a common language across a diverse research team and allowed a degree of abstraction from specific empirical findings.

Using NPT presented some challenges; coding interviews and previous papers according to NPT constructs was not straightforward, as there was some overlap between the constructs, and some uncertainty about how they manifested empirically [39, 44, 56]. The challenges were partly due to the slightly different purpose of the interviews included in the secondary analysis, which focused on specific SSME programmes rather than the broader question of routinisation in a wider system. Incorporating insights from previous papers using NPT in primary care was also complicated by the different ways in which they had applied NPT, and the varying levels of detail presented in the papers. A further challenge was ensuring that our application of NPT captured data that did not obviously fall into one of its constructs; to address this our teamwork approach to analysis enabled discussion of such data and in doing so helped develop our shared meanings.

Towards the end of phase 1, we moved from explicitly drawing upon NPT (and presenting findings according to NPT constructs) to organising our findings into four domains driven by data specific to our focus, i.e. the attributes of an embedded SSME. We made this change to ensure the language used to describe the process was accessible to wider stakeholders, made intuitive sense and had face validity as we moved towards the consultation work involved in phase 2, and to ensure the ensuing discussions moved from the broader challenge of normalising complex interventions *in general* to the challenge of normalising SSME *specifically*. This meant moving away from NPT as an organising framework (though it remained implicit in the attributes themselves). Consequently, the attributes were prioritised and ranked for importance by wider stakeholders without reference to the four domains of NPT. In the event, the prioritised attributes that were taken forward as essential components of the intervention did largely map onto NPT’s four domains (for example, see Table 5). Our data tables provide an insight into the mapping processes that we undertook, which enabled tracking throughout all phases, from the analysis of the data and the NPT framing of it through to the eventual intervention components. Our approach is in line with NPT authors’ proposed use of NPT as a tool to be used flexibly [62]. Nevertheless, if we assume that all four NPT domains are equally important for the long-term prospects for normalisation and routinisation of an intervention, then arguably it might be preferable to use them explicitly to organise a proposed intervention throughout the development process – perhaps in simplified form to ensure accessibility to wider stakeholders.

### Learning from a phased approach to intervention development

By using NPT as a theoretical framework for integrating insights from the literature, from rich existing datasets and from consultation with a wide range of stakeholders, our approach sought to follow best practice in intervention development [18]. It ensured the intervention was informed by abstract, generalised and concrete, local forms of knowledge, and that the views of all relevant stakeholders were included. The active involvement of stakeholders in Phase 2 was key, given the lack of ‘voice’ of some groups in the data assimilated in Phase 1. Their involvement ensured that key influences were addressed, and helped us to understand the contexts in which stakeholders understood and made sense of SSME – including not only day-to-day decisions about referral and uptake, but also the less visible work of groups such as commissioners, whose influence on funding, governance and mechanisms for encouraging uptake is crucial. Throughout the three phases, regular ‘back and forth’ meetings between the team responsible for analysing data to inform development and the team responsible for designing the intervention helped to ensure the resulting intervention was rooted in evidence. It also meant that the toolkit components of the intervention could be enriched by case studies and examples from the data.

There is learning from our approach which could be useful for future intervention design. While Phase 1 paid specific attention to the views of patients via both the use of existing evidence and through PPI representation in Phase 2a (and throughout the wider programme of research [16]), there may have been merit in further patient input in Phase 2b. The prioritisation of attributes during Phase 2 into the most important components that ultimately informed the design of the intervention in Phase 3 depended crucially on what stakeholders thought to be most pressing, relevant and realistic in designing an intervention. The stakeholder consultation process therefore added a crucial sensitivity to the realities of SSME delivery in their local contexts, but arguably it also meant that the design of the intervention was constrained by prevailing perceptions of what was feasible. Thus, the pragmatism offered by stakeholders during Phase 2 reined in and focused the intervention in ways that may equally ensure a pragmatic focus on what is possible, and limit the ambitions of the intervention to the most immediate preoccupations and pressing concerns. Certainly, the intervention does not address certain system-level issues facing SSME, such as limited capacity and resources, competing priorities, and divergent incentive frameworks for different actors in the system.

## Conclusion

Our paper describes the process through which an intervention to improve referral and uptake rates for self-management education for patients with T2DM – the Embedding package – was developed and designed. Our approach to intervention development sought to incorporate insights from a range of perspectives, and balance generalisable findings from existing sources with insights from stakeholders with direct knowledge and experience of SSME and theory on implementation.

In describing the development process and offering reflections on its strengths and limitations, we offer learning that we hope will be helpful for others seeking to use existing data sets as part of a phased approach to intervention development, or those seeking to use NPT to guide this process. NPT as a theory offers a framework for making sense of the mechanisms vital to normalisation. Our intervention-development process provides a way of operationalising these mechanisms in a specific area of practice, and identifying plausible, acceptable, stakeholder-consulted ways of realising normalisation. What neither of these things offer is any guarantee that the selected components will work in the way we anticipate, or that the whole intervention will be equal to or greater than the sum of its parts. Rather, they must be followed by piloting, adaptation and thorough process and outcomes evaluation.

## Supplementary Information

**Additional file 1: Supplementary Table 1.** Key constructs of Normalisation Process Theory (21,24). **Supplementary Table 2.** Example extracts from literature coded to NPT framework.

## Data Availability

The data that support Phase 1a of this study were made available by the investigators of the original studies to the research team for the sole purpose of secondary analysis. The data that support Phase 2 of this study are available from the corresponding author (HE) upon reasonable request and with permission of both the corresponding author and the Chief Investigator (MJD).

## Footnotes

1 It should be noted that the interventions of focus in the literature review were not necessarily SSME; rather, they were a range of interventions in primary care which had been developed or evaluated using NPT.
