# Supplementary material for: Using Normalisation Process Theory (NPT) to develop an intervention to improve referral and uptake rates for self-management education for patients with type 2 diabetes in UK primary care
Source: BMC Health Serv Res. 2022 Sep 27;22:1206. doi: 10.1186/s12913-022-08553-7 (PMC9513934; doi:10.1186/s12913-022-08553-7)
Supplement: Supplementary file 1 — Additional file 1: Supplementary Table 1. Key constructs of Normalisation Process Theory (21,24). Supplementary Table 2. Example extracts from literature coded to NPT framework. [file 12913_2022_8553_MOESM1_ESM.docx]

**Supplementary Table 1: Key constructs of Normalisation Process Theory (21,24)**

| Coherence | Participants distinguish the intervention from current ways of working |
| --- | --- |
|  | Participants collectively agree about the purpose of the intervention |
|  | Participants individually understand what the intervention requires of them |
|  | Participants construct the potential value of the intervention for their work |
| Cognitive participation | Key individuals drive the intervention forward |
|  | Participants agree that the intervention should be part of their work |
|  | Participants buy into the intervention |
|  | Participants continue to support the intervention |
| Collective action | Participants perform the tasks required by the intervention |
|  | Participants maintain their trust in each other’s work and expertise through the intervention |
|  | The work of the intervention is appropriately allocated to participants |
|  | The intervention is adequately supported by its host organisation |
| Reflexive monitoring | Participants access information about the effects of the intervention |
|  | Participants collectively assess the intervention as worthwhile |
|  | Participants individually assess the intervention as worthwhile |
|  | Participants modify their work in response to their appraisal of the intervention |

**Supplementary Table 2: Example extracts from literature coded to NPT framework**

| NPT constructs | Selected example data |
| --- | --- |
| **Coherence** | *Understanding and differentiation of the intervention from routine care*   - [The intervention] was perceived as potentially providing something distinct to current services, and delivery ‘closer to home’ was considered to be ‘much less daunting’. (39) - The CMs did find that the BA intervention encouraged them to develop joint plans with patients to a greater extent than in their usual practice. (40) - In terms of making sense of the new innovation there appeared to be little differentiation made between self-management support and the [‘the intervention’] approach from normal practice. (41) |
| **Cognitive participation** | *Engagement with the intervention*   - Professional buy-in to the concept of providing [the intervention] for people with severe COPD was evident. Members of the community teams described having attended ‘introduction to palliative care’ courses or perceived their role to include advanced care planning. (43) - Practice buy-in to the concept of using the [the intervention] tool for supporting the self-management process was evident … The main driver of the intervention within the practice was the nurse, as it was he/she who would deliver the intervention. Their pre-study enthusiasm was apparent, as they perceived their role in asthma management to include self-management advice and action planning. (47) - The mode of delivery of [the intervention] was considered to be key to their potential value because stakeholders were well aware of the challenges that present, especially when trying to get busy GPs on board. For example, stakeholders in England were concerned about getting GPs to commit to a full day of training and a GP stakeholder in Greece reported real concerns about fitting training into his/her schedule. (52) |
| **Collective Action** | *Undertaking the work of the intervention*   - Not briefing locum doctors (and it was latterly revealed new salaried GPs too) or notifying the study team that new staff potentially needed training meant that the intervention was not fully communicated and “collective action” (thus delivery) was not always achieved. (53) - Components designed to focus the whole general practice’s attention on [the intervention] were frequently reported as successful. Computer prompts were reported by many as important in helping staff remember to make the offer. (57) - Some respondents described the impact of external constraints on their capacity to implement the skills and expertise of generalist practice: “You’ve always got the targets coming out at you from the computer ... I think the problem is time.” (Interview group, experienced GP). (44) |
| **Reflexive monitoring** | *Understanding and evaluating the work of the intervention*   - Some GPs did report receiving positive feedback from the patients about their experience with the CMs and of the intervention (BA, MM), which led the GPs to believe there was some value in the intervention. (44) - Patients believed that the intervention made them pay greater attention to their asthma/health, affirming the importance of good self-management and motivating them to take an active part in managing their health. (47) - There was a perception of a mismatch between the monitoring required by external systems and that to support EGP. With a sense that those outside of General Practice failed to understand or value the EGP approach (only 19% of survey participants thought that their Primary Care Trust thought EGP was worthwhile). (57) |
